# The Perfect Postnormal Storm: COVID-19 Chronicles (2020 Edition)

**DOI:** 10.1177/19467567211027345

**Published:** 2021-06

**Authors:** Christopher Jones, Jordi Serra del Pino, Liam Mayo

**Affiliations:** 1 Walden University, CO, USA; 2 Blanquerna, Universitat Ramon Llull , Barcelona, Spain; 3 University of the Sunshine Coast , QLD, Australia

**Keywords:** postnormal analysis, futures studies, manufactured normalcy fields, postnormal shifts, levels of uncertainty/ignorance, postnormal burst, lag, and tilt

## Abstract

This essay addresses the COVID-19 pandemic as a case study in postnormal times phenomena: a perfect postnormal storm. The essay introduces basic concepts of postnormal analysis and provides examples of the acceleration of speed, scope, scale, and simultaneity of change in a number of human and natural systems and gives examples of the accelerating complexity, chaos, and contradictions that characterize phenomenon and systems as they become more postnormal. The related concepts of the layers of ignorance and uncertainty are explored related to the movement of phenomenon toward postnormal states. The importance of the idea of manufactured normalcy fields and resistance to or accommodation of postnormal burst, such as lag and tilt, can help to better understand the postnormal landscape. For example, the pros and cons of returning to “normal” raise fundamental questions about the logic and wisdom of the dominant growth and economic paradigm.

## Introduction

Ziauddin Sardar welcomed us to postnormal times a decade ago in a germinal 2010 article that proposed a new theoretical approach to provide a better understanding of how change is unfolding in the 21st century. Sardar’s initial description of postnormal times theory generated substantial interest within the futures studies field (Gary 2010; Markley 2011, 2012; Montuori 2010; Ringland 2010) and criticism (Cairns 2017; Cole 2010; Cubbit, Hassan, and Volkmer 2010; Gidley 2010; Kapoor 2010). Sardar (2015) responded to criticisms and, further, presented a timeline of how various civilizational artifacts such as meaning, truth, knowledge, world order, and governance have been transformed over time from the classic period to modern, postmodern, and then to contemporary postnormal times. Sardar and Sweeney (2017) further developed the concepts, exploring the temporal topography and possibilities of change over time. A *Postnormal Times Reader* emerged in 2017 with 20 new and reprinted articles that added to the postnormal times body of analysis and knowledge. Postnormal times analysis has been further explored by futurists and others across a range of subjects and disciplines: agriculture (Coventry 2015); art and design (Van Leemput 2018); conservation biology (Colloff et al. 2017); creativity (Montuori 2010); education (Çepni 2017); epistemology (Mayo 2020); evaluation (Schwandt 2019); futures studies practice (Fuller 2017); global change/weirding (Jones 2019; Sweeney 2017); intelligence services (Serra and Sardar 2017); Islam (Muzykina 2018); science education (Gilbert 2016); and sociology (Bussey 2018). Over the past few years, the number of events, issues, and cases that support postnormal times theory have grown rapidly. We argued in a 2020 blog series^1^ that the current COVID-19 pandemic and ripple effects are a perfect example of emergent postnormal times phenomena.

## What Happened to “Normal?”

Sardar (2010) characterized postnormal times as “an in-between period where old orthodoxies are dying, new ones have yet to be born, and very few things seem to make sense” (436). Bauman and Mauro (2016) put it this way: “We are hanging between the ‘no longer’ and the ‘not yet’ and thus we are necessarily unstable” (20). We are thus living in “a transitional age, a time without the confidence that we can return to any past we have known and with no confidence in any path to a desirable, attainable, or sustainable future.” A common and persistent meme of the pandemic in the mass media, press, and social media is “when will we get back to normal?” Everything has been disrupted by the pandemic, but is a return to normal even possible or desirable?

It seems clear to us that the accelerating rate of change in contemporary times has had something to do with it, as has our ability as individuals to communicate with millions of people at the speed of light due to the spread social media. We live in a globalized world that is interconnected and interdependent in numerous ways. News and information, as well conspiracy theories and misinformation, spread at astonishing rate; we are primed to act and interact in an instant. All these actions, interactions, and interconnections at every level, from local to global, at nearly every moment of our lives, constantly and perpetually, generate a change that is outside of previous human experience: postnormal change. A convenient way to think about it is to consider the four letters S of change: the speed, scope, scale, and simultaneity of change (4Ss). The overall acceleration of change is a direct product of combined force of the *speed* with which change occurs; the global *scope* of this change; the fact that this change can *scale* down to individual levels and scale up; and that these aspects of change occur with increasing *simultaneity*. The 4Ss define the dynamics that generate postnormal change. The postnormal times conditions are both part and parcel to the emergent COVID-19 pandemic.

Postnormal change does not intrinsically produce postnormal phenomena, but in an interconnected, globalized world, with a multiplicity of scales, accelerating speed, scope, and simultaneously interacting elements with nonlinear feedback loops, the consequences manifest in complexity, chaos, and contradictions (the 3Cs). *Contradictions* come to the fore and enhance the *complexity* of social, technological, and economic systems. These systems are wholes far greater than the sum of their parts; they exhibit properties of emergence and cannot be analyzed in terms of their parts but only be understood in complete, unabridged form (think: hyperobjects, Morton 2013); and the myriad interacting components self-organize to produce new patterns and structures. Complexity and contradictions then generate positive feedback loops leading to *chaos*. It is when the complexity, contradictions, and chaos emerge together that postnormal phenomena become visible.

Together the 4Ss and the 3Cs constitute the basic pillars of the postnormal times theory and are complemented by two other aspects: rising levels of resulting *uncertainty* and *ignorance* (see: Sardar and Sweeney 2017), which vary in kind, but generally grow in tandem as postnormal phenomenon develop and mature. Over time, the extent of ignorance and uncertainty can expand or deepen dramatically. Over longer time frames and across greater scales of change, ignorance, and uncertainty can accelerate. Postnormal phenomena generate and are deeply embedded in growing uncertainties, which in turn produce a variety of ignorance. We use the *three tomorrows* scenario planning and scenario building approach (addressed in other articles and essays in this issue), each of which has a particular type of ignorance and uncertainty, as described by Sardar and Sweeney. Three tomorrows are where futures studies meet postnormal times analysis in attempting to explain postnormality. We argue that to deal with uncertainties and gaps in our knowledge, and we need to expose our individual and cultural biases. We have to consider uncomfortable, unthought, and/or unimagined futures, to help re-examine our basic assumptions, ideas, values, narratives, and worldviews. We need to show humility: postnormal events and issues cannot be controlled or managed—they can only be navigated.

The COVID-19 pandemic is a postnormal phenomenon/hyperobject because it satisfies all of the postnormal criteria: complexity, contradictions, and chaos, and their handmaidens—the speed, scope, scale, and simultaneity of accelerating change.

### Speed

The contagion spread incredibly fast. The first confirmed case was in 17 November 2019; 5 months later, there were 2.5 million confirmed cases and more than 167,000 deaths. At the time of this writing, global cases were reportedly 26.4 million, with 870,000 deaths.^2^ Given the shortage of test kits, undercounting early in the pandemic, and data collection and reporting inconsistencies, the real figures are probably much higher. Similarly, the economic impacts were swift—US equity markets lost roughly 40% of their value between mid-February and late-March 2020; the technology-heavy Nasdaq market regained most of that value in 2 months. These were rapid and historic shifts. Lockdown policies had almost immediate consequences for employment: US unemployment numbers doubled from 3.3 million to 6.6 million in the third week of March.^3^ Misinformation and conspiracy theories also spread at the speed of light. Social media have played a central role in accelerating the speed of active responses to video records of police murders and brutality. Protests have occurred in the wake of the pandemic, both on the right and left, but most notable are the protests against police brutality in the United States and related anticolonialism protests, internationally.^4^ The pandemic itself: it will be the fastest-growing global pandemic in human history. The ripple effects have moved equally fast.

### Scale

Global 2020 infection maps graphically demonstrate the spread, first from Wuhan, through land transportation systems, then globally thanks to air travel systems through clusters of infection: parties, ski trips, and conferences. Currently, only a single African country and geographically isolated Pacific Island countries have no detected cases. It is just a matter of time until they too report local infections. Thanks to globalization and interconnections, it will be the most widespread global pandemic in human history. The only continent spared thus far is Antarctica. In the United States, mid-West states that largely avoided outbreaks in March are seeing surges in cases as we write this.

### Scope

The combination of a comparatively high degree of infectiousness, undetected transmission by asymptomatic individuals, and our lack of knowledge about the virus made human confinement the best option to fight further spread. Some countries have been more successful than others, and intrastate and international travel continue to pose challenges to contain the coronavirus. The world’s economy and global supply chains have been under great stress. After starting in China, COVID-19 triggered cascading effects. The scope is so vast and immediate that threats to industrial capitalism and liberal democracy are potentially far greater than the 2008 global recession. Tens of thousands of small businesses and restaurants have closed in the United States,^5^ and likely multitudes more, globally. Industrialized nations initially spent billions of US dollars/euros on unemployment and wage/unemployment subsidies. There are possibly serious downstream consequences for future generations burdened with the debt incurred. Or will debt simply be forgiven, in national and international jubilees? While it is impossible to predict the outcome, the scope of these disruptions will echo through the lives of young people today. Reconceptualizing, or reforming, the global market economy, is perhaps one of the main outcomes of this pandemic. It has had an impact on almost every individual, in every community across the planet; there is no telling what the mid- or long-term effects will be. The World Health Organization (WHO) projects that a widespread COVID-19 vaccination not likely to be available until the middle of 2021.^6^

### Simultaneity

The pandemic has altered billions of lives. Initially, communities learned how to live indoors, and during the early 2020 lockdown, many cities looked like ghost towns. Wildlife crept back into urban spaces and nature had a short reprieve from human activities. Control of the coronavirus spread was effective in some places that led, in late spring, to cautious reopening. The rules were often ignored. There were notable successes, but simultaneous nonconformity—particularly risky behavior by adolescents and young adults—that caused outbreak clusters and resurgence into the summer of 2020. Meantime, global supply chains were disrupted and the shortcomings of production and distribution of personal protective equipment (PPE), much of it manufactured in China, raised questions about reliance on global distribution systems. Retail and manufacturing similarly suffered, and many businesses have restructured to use local resources and suppliers.^7^ New wrinkles in our reliance on globalization were easier to see. A new set of economic relationships may be unfolding and beg the question: How much of our present arrangements will survive and recover after COVID-19 runs its course, assuming we can achieve widespread vaccination? Will the direct short-term impacts on the economy and the rippling secondary and tertiary impacts, to which we are not currently paying attention, transform economic and technological systems? What will the longer-term consequences be of joblessness, isolation, masking, economic disruption, housing, generational (age cohort) impacts, education, travel, and transportation realignments? Telecommunications technology, the internet, and instantaneous communication provide greater awareness and ignorance about all of these things, at the same time.

Arguably the COVID-19 pandemic has all of the expected characteristics of postnormal change, but what made it a postnormal event? The 4Ss describe the changing nature of change itself, but Sardar’s original study argued that postnormal times emerge as a result of the resulting and growing complexity, chaos, and contradictions within human systems—the 3Cs. More than being the driving forces or characteristics of postnormal times, they are also postnormal enablers—intrinsic factors that need to combine or overlap in order to trigger postnormal phenomena. Complexity, chaos, and contradictions have always been a factor of life, but they are converging and feeding each other now in human social and technological systems in ways that are unique, unpredictable, and increasingly nonlinear.

## Complexity

Complexity is the property of a system that has multiple components that interact in many ways. Complex systems exhibit behavior based on the interaction of these components. Some properties that complex systems feature include: self-organization, nonlinearity, emergence, feedback cycles, and adaptation. Growing complexity will require a better “understanding of the dynamics of intertwined human and planetary systems” (Schultz 2016, 330). To grapple with the postnormal aspects of complexity, consider plurality of diverse elements in the COVID-19 system and their interconnections. The pandemic was a result of several elements acting synchronously in response to the emerging coronavirus. First, a large Chinese diaspora spread across the world after 1850. Second, the timing of the emergence of the pathogen coincided with the Chinese lunar New Year. Third, continuous national and international travel and transportation systems. Those latter systems spread the virus at astonishing speeds. Fourth, the pandemic has had impacts across a whole range of sectors of the economy: from international finance to health services, employment, food production, and manufacturing. The pandemic has exacerbated system stress by restricting travel and freedom of movement upon which the systems originally depended. Business and public organizations have adapted, and there has been some cost savings for corporations, but the use of Zoom meetings and remote employment contribute complexities of their own.

Over Chinese New Year 2020 celebrations in January, millions of Chinese traveled from the far corners of the Earth to return home to celebrate with families, in what is among the largest annual population movements across the planet. Diffusion maps of the virus across China show how widespread and complex air, rail, and road transportation made viral transmission unavoidable. This tapestry of multiple interconnections made the spread of the virus inevitable, despite the apparently effective lockdown measures in Wuhan, and the surrounding region, because the virus was already loose in the world. The spread in the Americas was primarily via Europe and followed a similar diffusion pattern of clusters, super-spreader events often involving long-distance travel. The outbreaks in eldercare facilities obscured the fact the young people can be asymptomatic hosts.

Complexity, as a feature of modern life was a priori, a given, well before the pandemic. One of its emerging lessons may be that fragmented, self-governing political systems are poorly adapted to a planetary civilization. The liberal democratic concept of personal liberty may be incompatible with maintaining public health. To make matters worse, the global system is not even close to a system of governance, it is still a Wild West of nation-states not unified, but separated by territorial integrity, tenuous sovereignty, and a lingering attachment to the Peace of Westphalia. With roughly 200 separate countries, not to mention the thousands of cities, states, and territories in the mix, connectivity is sought even more desperately to respond to the scientific, economic, social, and political needs of the pandemic and the problems/challenges it will generate in our futures.

Another factor adding to global complexity is China’s growing financial muscle and status as the world’s second largest economy and growing military power. What will economic contraction mean for its Silk Road Initiative and infrastructure projects around the world? In any case, system complexity has been fueled by the success of the Chinese economy and the huge demographic shifts from rural villages to megacities. China has become an increasingly mobile society. Making things even more complex, China has become a critical player in the global supply chain. Before the pandemic, China produced more PPE than the rest of the world combined. Since February, China’s output has increased five-fold and it dominates the PPE market.^8^

The concurrent New Year’s celebration and mass travel increased system complexity. The complex mix of unitary, federal, and confederal states, the WHO, and leading experts often took (continue to take) disparate responses in restrictions of movement and epidemiology tracking. China is criticized for taking draconian steps, while South Korea is praised for taking a democratic but communitarian response. When we wrote our blog in May, Italy, Spain, and the United States seemed to respond “too little, too late” to avoid serious casualties. The United States is now the record holder for the highest caseload, over 11 million, and nearly 250,000 dead. The United States, Spain, Iran, and parts of Latin America remain hotspots at this writing.

## Chaos

The second C in postnormal analysis is seen as the feature of dynamic nonlinear systems that exhibit disproportionate inputs and outputs; the COVID-19 pandemic has shown chaotic behavior in many ways. Indeed, the fact that we know so little about the virus has not helped, but its high rate of infection (R0) and asymptomatic carriers have resulted in a wide range of responses leading to a large variance in results. Differences in geography, climate, and community infections have continued to make it hard to identify patterns. It is increasingly clear, however, that asymptomatic transmission by younger people is prevalent,^9^ how the virus spreads, but the long-term consequences of the disease are troublesome. For example, long-term pulmonary and coronary complications affect many survivors^10^ along with brain fog, circulation problems, and other side effects of the disease. It remains to be seen what the longer-term medical and healthcare needs will be for people, especially young people, who survive with deeper underlying damage to their bodies. The pandemic has been like an event where thousands of butterflies begin to fly simultaneously, without anyone noticing them, and they then unleash a series of hurricanes far too powerful to be mitigated.

Chaos was particularly evident in the first 2 months of the global pandemic, with the initial reluctance of China to accept the Wuhan outbreak, then immediate lockdown. The lack of science and knowledge led to mixed messages about mask effectiveness, and WHO officials did not always appear to agree with nation-state spokespersons. The intricacy (complexity and contradictions) of systems and messaging of surveillance and communication channels and media were revealed. The overlay of social media complicated matters more, with confusion about conspiracy theories, basic facts, and then presidential and prime ministerial fake news. The EU came to face the realities of decisive leadership on one hand, and the re-emergence of hard borders, on the other hand. Sovereign decision-making and reliance on supply chains hampered manufacture and distribution of basic protective gear, and leaders sent mixed messages to consumers about appropriate behavior. Vaccine production has also been compromised by short-term capitalism and a lack of strategic, long-term responsibility for the possibility of pandemic. Ironically, contagion war gaming and role-playing has been widely used in academic and foreign policy settings, but apparently no one at the top paid attention or cared enough to respond in time. Response to the pandemic reflects the complexity of the global milieu. For example, in the United States, leaders at the local level, governors, and city mayors have been making the more aggressive and effective science-based policies to prevent the spread of the coronavirus (like in Italy, where some mayors have personally enforced the confinement). There has been widespread criticism of the US president for a lack of consistent and effectively leadership in responding to the crisis—his campaign continues to refer to the pandemic in the past tense,^11^ despite the growing case numbers, appearing to hope that the whole thing will just go away!

## Contradictions

The contradictions in the wake of this crisis are obvious. Efforts by politicians to downplay the crisis and avoid panic in many cases have made it worse. Long held values get in the way; the values of economic production, jobs, and continued growth contradict community health and physical well-being. The pandemic will illuminate, like no other, the direct relationship between androcentric values, particularly economic values, and the rest of the planet. Preliminary figures already demonstrate the improvement in the quality of air, water, and atmosphere due to the economic slowdown. COVID-19, some say, may be Mother Earth’s rejoinder.^12^

The pandemic brings a host of other contradictions to the forefront. It has been driving a centrifugal globalization dynamic, but forcing a centripetal, inward spiral with travel restrictions, surge lockdowns, isolation, safe distancing, and masking. Some are even calling for economic deglobalization in a “waning hyper-globalization era.”^13^ The question is, while some countries seem to be doing well, so far, will they be able to make it through a global recession or depression? Projections for a fall resurgence in the Northern hemisphere are dire, not only for the pandemic but for the growth of hunger and homelessness barring more economic stimulus and/or unemployment compensation (now at a standstill in the United States). A global food crisis is emerging.^14^ Social distancing reinforces the importance of close-community networks, yet it is also lethal for local retail as it lacks the structure to deliver, while Amazon and the like are making record profits.^15^

The pandemic may provide growth opportunity for some sharing economy firms (Globo, Uber, and Airbnb), on the other hand, the impact on gig economy workers is less clear. Unemployment may drive more individuals into the sharing and gig economies.^16^ The new business models may suffer the consequences of riders and drivers getting sick, homeowners going bankrupt, and the vicissitudes of the general economic and employment crisis. The pandemic calls for effective and inspiring public and private leadership, leadership that has been sadly lacking (e.g. Greta Thunberg’s scolding of world leaders on global warming^17^) and characterized by fructuous ideologues worldwide (with few dignified exceptions) that enable or encourage authoritarianism and the rise of strong men who go unchecked. The internet and telecommunications now make it possible for people to stay in touch with friends and family near and far, and for many professionals to carry on more-or-less “as usual.” But it will also accelerate growth of cyber-infrastructure, the automation process, and will likely leave millions unemployed. Perhaps the most poignant contradiction has been moral dilemma, the tension, between saving lives or saving jobs. Or even worse: killing people that do not respect confinement (a measure originally designed to keep them safe) as in the case of President Duterte of the Philippines who ordered lockdown violators be shot.^18^ As shocking or worrying the emerging pandemic contradictions may seem to be, the main lesson is that the contradictions only increase the postnormal nature of the phenomenon. How are we going to cope, as individuals, communities, and societies as things become more and more postnormal?

The staff, fellows, researchers, and directors of our Center^19^ are particularly concerned and disturbed about the likelihood that the COVID-19 will have its greatest impact on the most vulnerable and marginalized people on the planet; our primary concern is decolonizing futures (see Sardar 1999). In industrialized countries, it is clear that the elderly and marginalized are expendable. There appears to be great media attention and public gratitude to “first responders” and yet we collectively and our leaders are allowing hundreds if not thousands of nurses and doctors and other healthcare workers succumb to this pandemic. The general public might not have been able to foresee COVID-19, but environmental scientists, epidemiologists, and other experts as well as futurists forecast the inevitability of zoonotic pandemics to follow in the wake of MERS, SARS, swine flu, and Ebola. However, now that the event has begun to unfold as a postnormal phenomenon (we may not even be halfway through the pandemic at this point in time), we need to learn to navigate it. Of course, the best way to navigate a storm is to be able to anticipate and find the course that can take us away from it, and this is why futures studies are important. How can we navigate this crisis? How much do we not know about current COVID-19 crisis? And how are going to deal with our ignorance?

## Navigating the Pandemic

What is known about SARS-CoV-2? Researchers appear to know where it originated,^20^ how it spread, and have a rough idea of its contagion levels. We know that the spread of the coronavirus has been matched by the spread of information—and misinformation—about the virus and the disease COVID-19 through social media networks, which have exacerbated levels of anxiety and clouded clarity in decision-making at every level. We know that governments have made decisions to lock down communities to enact social distancing to mitigate against viral spread, while the business sector suffered the loss of customers and workers, and many industrial and service sectors have been damaged. Tourism and the travel industries have been hard-hit, as have retail trade, but impacts in other sectors others are minimal or mixed.^21^ The global economy is in decline yet global equity markets have seen both volatile lows and recent highs that seem illogical in the face of economic uncertainty. Healthcare workers, across the globe, are now on the front line of the gravest existential threat to humanity in a century. Global warming and social inequity movements have been eclipsed by the global pandemic.

How can individuals and organizations better navigate the emergent global COVID-19 crisis? We suggest that it is essential to address these two questions: (1) How much do we not know about the COVID-19 pandemic and its consequences at this point in time? And depending on the answer to that question: (2) How are we going to act based on determining what we do not know but still need to find out?

## Manufactured Normalcy Field

Cognitively, human minds excel at normalizing whatever happens. Our brains tend to reject contradictions and anomalies when we experience cognitive dissonance. It is an evolutionary biology advantage that allows individuals to adapt quickly to external change, but it becomes a hurdle when one needs to be open to a wide range of possibilities, particularly novel ones. Rao’s (2012) notion of a cognitive *manufactured normalcy field* aligns with postnormal adaptation to the impact of normalcy in our thoughts and behaviors. The manufactured normalcy field is the ontological construct, a hyperobject, that reaffirms normalcy despite disruptive external change. The manufactured normalcy field is not intrinsically positive or negative, but an adaptive strategy that may need recalibration in the face of rapid change or the accumulation of uncertainty and ignorance in postnormal creep. The manufactured normalcy field tends to reinforce conventional linear thinking and induction as the best strategy to deal with the “normal.” COVID-19 entails deeper layers of uncertainty that cannot be overcome with plain ignorance. The normalcy of concerts, spring break, teen parties, bars, and large weddings in many societies is challenged by recommendations or mandates to wear masks, social distance, and the new norms of public health policy.

## Postnormal Shifts—What the Shift?

Postnormal theory has argued that the greater the influence and convergence of complexity, chaos, and contradiction within a phenomenon, the greater the uncertainty. Yet, uncertainty is not unidimensional, simply by increasing in size, rather as the 3 Cs overlap each other, uncertainty grows in phase changes: *postnormal creep*. Postnormal creep is the specific process any event or phenomenon follows when developing its postnormal potential and has a material aspect (uncertainty) and a cognitive aspect (ignorance). The more postnormal creep progresses, the greater uncertainty becomes and depending on the degree of uncertainty, our individual ignorance becomes measurably deeper and/or wider. Once postnormal creep reaches a certain threshold, there can a *postnormal tilt*, a readjustment to the manufactured normalcy field, or a phase change: a *postnormal burst*. We argue that COVID-19 has features of all of those manufactured normalcy field adjustments and is multilayered with cross-sectoral, temporal (old and new characteristics), and cultural adjustments. The postnormal creep and adjustments are widespread in the emergence of SARS-CoV-2 and the resulting COVID-19 pandemic, which provides further proof that COVID-19 is a postnormal “perfect storm.” Arguably, the global pandemic, considered as a hyperobject, has become a postnormal burst.

## Depths of Uncertainty

Continuing to unravel the effects on the manufactured normalcy field, postnormal time theory describes layers of uncertainty from shallow to deep. Challenging this normalcy are the underlying driving forces of change in the 21^st^ century, the tsunamis of change (see Dator 2009) including demographics, economics, globalization, technology, and the environment/climate change. These tsunamis are the underlying dynamics accelerating global change. What propels any major force into postnormal space is the accelerating speed, scope, scale, and simultaneity of changes to those forces and the concomitant complexity, chaos, and contradictions that follow. The driving forces and postnormal dynamics demonstrate a consistent pattern of creep. How creep leads to burst is best understood through the interplay and combination of growing degrees of ignorance and uncertainty.

Uncertainty in postnormal theory and analysis is a measure of our capacity to realize what is going on, both quantitatively and qualitatively. Uncertainty also builds, following the trajectory of the postnormal creep. In the case of SARS-CoV-2, researchers knew very little at the beginning, but pundits and some political leaders seemed to assume it would be like any other coronavirus and did not express concern about it. At this point, leaders and individuals faced *surface uncertainty* and most people assumed that our accumulated knowledge would carry us through the outbreak. Public leaders could use what was learned in the flu pandemic of 1912, or perhaps the polio epidemic. Very soon though, the novel coronavirus was understood to be far more aggressive and more lethal than had been thought.

SARS-CoV-2 behaved in unfamiliar ways and it took time for researchers to uncover the mysteries and quirks of the coronavirus, as a pathogen: by then the progressive postnormal creep moved into deeper territory: *shallow uncertainty*. Some observers wondered if the pandemic and the resulting economic crisis might shake the very foundations of modern institutions or question collective assumptions about globalization, capitalism,^22^ of institutions like the EU,^23^ and the idea of materialistic growth itself.

As human societies plunge deeper into the pandemic, we may need to ponder if humanity will sink even further into a state of *deep uncertainty*. Because the pandemic has occurred in the midst of already emergent postnormal phenomenon, the creep contributes to the existing depth of uncertainty about accelerated global warming, growing wealth and equity imbalances, and a host of other hyperobjects and wicked problems that threaten civilization or human survival. Social justice movements (e.g. Black Lives Matter) and the growing power of women (e.g. #Me Too) have similar transformative potential and could be accelerated or dampened by the pandemic. Other uncertainties abound: discoveries about the ubiquity of microplastics in the environment and plastic pollution were serious before 2020, but the demands for PPE and safe food handling have resulted in a dramatic growth in plastic uses.^24^ The consequences of this development and myriad others create ever greater uncertainty about the health of the planet and environment. Some of the concerns are existential (see Ord 2020). Assessing the kind of uncertainty humanity faces is just part of the equation: postnormal times theory posits that individuals and organizations should evaluate how that uncertainty is measured and processed and then plan how/work to compensate.

## Ignorance

Also important are the depths of ignorance that result from growing uncertainty. In postnormal times theory, the layers and depth of uncertainty are mirrored by the depth of our individual and collective ignorance. Ignorance is not only what it is that we do not know but also what we ignore. It is the cognitive side of postnormal creep and it grows to/corresponds with each degree of uncertainty. Each level of uncertainty aligns naturally with a level of ignorance. The levels of ignorance are as follows: plain, vincible, and invincible ignorance. Take surface uncertainty: although future outcomes may be unclear, decision makers should have a fairly a good idea of the direction outcomes may take and what kind of impacts are likely. In a state of surface uncertainty, previous experience really does help us to anticipate what might come next. Research indicating widespread coronary damage, even in nonhospitalized COVID-19 survivors, should lead us to expect to see greater incidence of heart problems and healthcare costs downstream. Researchers have learned from past pandemics and earlier crises and can gather relevant data, process useful information, and distill the knowledge to get society through the current crisis. This top level is *plain ignorance* and it is the cognitive approach humans are best at: mechanisms like linear thinking, dichotomy, induction, and specialization work beautifully here and give reassurance that knowledge can serve to reduce uncertainty. The pandemic cannot be really managed by business as usual or by “standard operating procedures.” Many Western cities had no contingency plans for a pandemic simply because they had no memory of one. At the beginning of the pandemic, cities and provincial governments may have believed they suffered from surface uncertainty but in fact were in shallow uncertainty territory.

Growing uncertainty, shallow uncertainty, required recognition of the deeper state of ignorance: *vincible ignorance*. New Orleans Mayor LaToya Cantrell never considered canceling or curtailing Mardi Gras^25^ in late February nor did US federal agencies raise concerns. The mayor knew how to respond to hurricane threats but not to COVID-19 spreading across the planet. Mardi Gras seeded Louisiana’s first wave of COVID-19. Plain ignorance was overwhelmed by uncertainty, and the mayor was facing vincible ignorance. The state of vincible ignorance demanded that policy makers address what was unknown. In strategic decision-making, nothing should be taken for granted. Accepting the assumption that COVID-19 was like a mild flu likely cost tens of thousands of lives.^26^ At the level of vincible ignorance, individuals and groups are forced to both acknowledge cognitive shortcomings and expand awareness by integrating all accessible and available knowledge. To respond to a pandemic, governmental responses cannot rely upon medicine alone but need to integrate epidemiology with health systems management, logistics, psychology, network management, and engineering. Response needs in the longer term may include resources or capabilities not even being considered currently. Because the lag in determining longer-term needs is central characteristic of vincible ignorance, leaders and public health officials have to accept that they lack the perspective of sufficient time to properly address the current situation. This appears to be where we stand with COVID-19 at this writing.

As humanity leans deeper into the pandemic, it seems increasingly likely that many lifestyle changes will be permanent, as the SARS impact in many parts of Southeast Asia and the acceptance of mask wearing. Some changes could go far deeper as economic disruptions continue to worsen as hunger and famine grow. The deep uncertainty component of the COVID-19 phenomena will require us to engage in addressing the last kind of ignorance, *invincible ignorance*. Invincible ignorance requires that we turn to our own epistemological structures, paradigms, narratives, and worldviews and ask if they are hindering our comprehension of the situation. Invincible ignorance is a kind of ignorance that requires that individuals examine the foundations of their worldviews to consider whether they are hindering our ability to grasp the Big Picture—the scope of the crisis and its consequences. COVID-19 seems, again, to be a perfect example of a postnormal phenomenon. If globalization dynamics boosted the spread of the virus, can/should we collectively or individually (boycott Amazon?!) try to change the dynamics? If present supranational structures have failed, we need to collectively develop new ones; if national governments cannot cope, they must be reformed or replaced.

At this deepest level of ignorance, it is not what can be learned from the pandemic experience but what we have to unlearn to better respond to the next pandemic (or: pick your environmental catastrophe). If current capitalist logic compels us to choose between saving people or saving the economy, then perhaps it is time to take a hard look at the extent to which the old ways (the old “normal”) were not sustainable or humane. As the imperfections of the system are laid bare, it may require our species to take a good hard look at our invincible ignorance deficits and not only imagine better futures but continue to work on realize wiser futures (see Sardar 2017). This level of ignorance also suggests that we need to anticipate and engage with unthought futures to explore the unknown unknowns, to consider wildcards and even catastrophe. Resilience will require thinking “outside the box.”

## Returning to Normal?

As researchers, we argue for a multilayered analysis of the levels of uncertainty in and ignorance about postnormal phenomenon and to apply the right kind of ignorance depending on the phenomenon being explored or scenario being built (see: 3 Tomorrows, particularly, this issue). The challenge is identifying which level of uncertainty to address and then apply the right depth of ignorance for analysis. This is a demanding challenge. The postnormal literature has dedicated considerable attention to what constitutes “normal”; it seems increasingly clear that when trying to sharpen our individual and organizational anticipatory capabilities, “normal” can be a big liability. Normalcy resists the consideration, and the wider use, of alternative future approaches; it even restricts what is acceptable in the present. A point made in a number of blogs and posts in the spring peak in COVID-19: “We will not return to normal because normal was the problem.^27^”

Maintaining a business-as-usual approach, in the face of accelerating change, is *postnormal lag*, when individuals and organizations persist in applying old recipes to new situations, while pretending that they work in spite of mounting evidence to the contrary (think: climate change denial). COVID-19 has shown several examples of this: every time a government has declared that there was nothing to worry about; or when they said that their health system was more than ready to face SARS-CoV-2; when they declared that the measure that worked in one place “would not work in our country;” when they kept stating that the country had already reached the peak (for days and days); and when they promised that their measures will keep the economy ready to go as soon as the confinement is over. Lag is one of the more dangerous aspects of the manufactured normalcy field, when individuals and organizations “bury their heads in the sand,” when the accumulation of anomalies in the Kuhnian (1970) sense push the paradigm toward collapse.

The continuation of postnormal lag can potentially lead to burst, to collapse, or transformation—a phase change. But before that, there is another possibility, an intermediate postnormal phase change: *postnormal tilt*. Postnormal lag may result from failed corporate and government leadership—not entirely new—but it appears that some leaders really do believe that decisions they make and directions they give are best in the absence of scientific and public health evidence. Moving beyond plain ignorance to acknowledge even deeper deficits and challenges to our knowledge, postnormal science is required to expand the boundaries of what we do not know and to expand our epistemological universe. Culture and governance differences provide different outcomes. China’s domestic COVID-19 strategy suggests that the postnormal lag can be beaten. Hong Kong, Taiwan, South Korea, Denmark, Germany, and Andorra took approaches to the early pandemic in effective ways, both in terms of public health and their economies. Either they had the capacity to see the real potential impact of the pandemic or realized that business-as-usual approach would not do.

The effect of a correction to the manufactured normalcy field is the postnormal tilt. It is modeled on the effect one feels when a modern bus stops at a curb and lowers hydraulically. The idea is that when the manufactured normalcy field can be altered in ways that we may actually experience the emerging postnormal nature of a rapidly developing event or issue. No matter how compelling or pervasive the manufactured normalcy field, humans do have the capacity to go beyond ontological and epistemological constructs and see phenomena for what they really are. (Or not: see the comments on object-oriented ontology and postnormal epistemology in this issue). The growing body count of the COVID-19 pandemic makes it painfully clear that adjusting the manufactured normalcy field is a very difficult thing to do.

Postnormal creep processes come to an end. According to Sardar and Sweeney (2017), this is *postnormal burst*, “when the system goes totally postnormal and there is no place to hide” (5). We have argued that the postnormal creep is expanding and extending and that tilt may have normalized the manufactured normalcy field somewhat. For example, working remotely and homeschooling are increasingly seen as the new normal. Larger systems are still behaving within the expected parameters of corporate capitalism and nation-state power appears to be reasserting itself (see Sardar in this issue). Although lifestyles have clearly changed, some of them likely to be permanent, there are still places to hide from postnormal times. Some organizations, and presumably systems, are adapting and thriving: high technology and space development growth continue relatively unrestrained.

When it fully arrives, the postnormal burst should both signal the end of postnormal creep and force the manufactured normalcy field to reset as the accumulated uncertainty is resolved, one way or another. The things researchers do not presently know will be discovered and resolved: the true SARS-CoV-2 infectiousness, including its incidence and virulence; its lethality; the COVID-19 pandemic’s total effect on the economy; and the actual impact of the pandemic on our lifestyles and our “normal,” ordinary activity. All these questions, so uncertain now, will become facts that we will be able to gather, measure, and process with plain ignorance. Even the remaining peripheral unknowns will fall under surface uncertainty. Right now, a burst might seem to be a bad thing. But it may also mean that individuals and organizations will become fully conscious of the situation, with little or no lag in the face of the new evidence, and we will gain experience and capacity to respond to future coronaviruses or other zoonotic outbreaks. Ebola, Zika, MERS, and SARS are reminders this is unlikely to be either the last or worst zoonotic pandemic. We may need to learn relatively fast, collectively, institutionally, and individually to respond to the next outbreak.

## Conclusion

The uncertainties, the ignorances, and the postnormal conditions driven by accelerating change have set the stage for unfolding postnormal phenomena. The COVID-19 pandemic has been a postnormal perfect storm because the pandemic has illustrated all of the key features and functions of postnormal times, and the creep and tilt that lead to eventual postnormal burst. The extent of socioeconomic and political changes in the future is addressed elsewhere (see: in this Special Issue) on three tomorrows of COVID-19. The pandemic has legitimized addressing taboo subjects and brought serious social justice and equity issues to the surface. The basic assumptions about liberal democracy, the rule of law, what constitutes truth and facts—all are under scrutiny.

For postnormal times analysis to have significance, the work will need to engage more fully and deeply with scenario building and planning, as we discuss in this issue with the three tomorrows approach. Organizations need to more fully engage in imagination, creativity, and envisioning preferred futures, such as the transmodern and now transnormal aspirations championed by Sardar in this special edition. We also need to more fully engage in the metaphysical and spiritual dimensions of postnormal times in order to come to grips with the meaning of these transformations (see Sardar this issue). The pandemic has caused great losses, to lives and livelihoods, and we need to come to terms with our grief and pain, to honor the people and activities we no longer have with us, but also celebrate the opportunity and gifts that have arrived unexpectedly.

Our intention here was to not only discuss the pandemic as a postnormal phenomenon but also to lay the groundwork for understanding the future monkeys of chaos, black swans, and elephants in the room, and proliferating jellyfish events that have been driving our social and technological systems to postnormal burst, prior to the current pandemic. Improving our collective understanding of postnormal times and the forces at work will be required to survive and even thrive in postnormal times. The challenges we are likely to face due to global warming may make zoonotic pandemics feel like one of the least of our problems. That could not be illustrated more graphically than the massive forest fires in the US Pacific Northwest at this writing. Multiple catastrophes are a likely harbinger of the future. The drivers of postnormal change are inexorably compounding complexity, chaos, and contradictions, further accelerating our ignorance and the uncertainty of it all. However, we now have tools to better understand and challenge the assumptions of the old normal, navigate emerging postnormal times, and chart a course to a more resilient, equitable, and wise transnormal civilization.

## Notes

1 https://postnormaltim.es/insights/postnormal-perfect-storm-part-1.

2 Retrieved 4 September 2020: https://www.nytimes.com/interactive/2020/world/coronavirus-maps.html.

3 https://www.vox.com/2020/4/3/21205830/march-unemployment-rate-data-misleading.

4 https://carnegieendowment.org/publications/interactive/protest-tracker?gclid=EAIaIQobChMIpd7-4-bQ6wIVx0XVCh15-gyJEAMYASAAEgLc9fD_BwE.

5 https://www.businessinsider.com/yelp-report-60-of-restaurant-closures-permanent-more-will-close-2020-7.

6 https://www.cnn.com/world/live-news/coronavirus-pandemic-09-04-20-intl/h_913a56fbb5c28940493ff6c4ebcf823f.

7 https://foodinstitute.com/focus/local-supply-chains/.

8 https://www.nytimes.com/2020/07/05/business/china-medical-supplies.html.

9 https://www.dw.com/en/coronavirus-clusters-linked-to-asymptomatic-younger-cases/a-54293037.

10 https://health.clevelandclinic.org/covid-19-can-also-damage-your-heart-not-just-your-lungs/.

11 https://www.vox.com/2020/8/26/21402124/rnc-coronavirus-past-tense-larry-kudlow.

12 https://www.onlynaturalenergy.com/avenger-planet-is-the-covid-19-pandemic-mother-natures-response-to-human-transgression/.

13 https://www.project-syndicate.org/videos/covid-19-deglobalisation-what-will-the-post-pandemic-world-look-like.

14 https://www.weforum.org/agenda/2020/05/global-food-chains-disruption-covid19/.

15 https://www.forbes.com/sites/jonathanponciano/2020/08/26/worlds-richest-billionaire-jeff-bezos-first-200-billion/#692397e74db7.

16 https://www.forbes.com/sites/jonathanponciano/2020/08/26/worlds-richest-billionaire-jeff-bezos-first-200-billion/#692397e74db7.

17 https://www.youtube.com/watch?v=RakpejJT6bc.

18 https://www.cnbc.com/2020/04/02/philippines-duterte-threatens-to-shoot-lockdown-violators.html.

19 https://www.cppfs.org.

20 For a skeptical view see: https://www.thenakedscientists.com/podcasts/naked-scientists-podcast/where-did-covid-come.

21 https://www.ibisworld.com/industry-insider/press-releases/ibisworld-monitors-the-coronavirus-impact-on-industries-around-the-world/.

22 https://www.weforum.org/agenda/2020/04/coronavirus-covid19-business-economics-society-economics-change.

23 https://www.city-journal.org/covid-19-european.

24 Retrieved 7 September 2020: https://www.ft.com/content/d5e27b1f-ba5a-4445-a329-1802cf70d619.

25 https://www.washingtonpost.com/national/coronavirus-new-orleans-mardi-gras/2020/03/26/8c8e23c8-6fbb-11ea-b148-e4ce3fbd85b5_story.html.

26 https://www.statnews.com/2020/06/19/faster-response-prevented-most-us-covid-19-deaths/.

27 https://www.odi.org/blogs/16815-covid-19-we-won-t-get-back-normal-because-normal-was-problem.
